# Impact of a Simple Educational Intervention on the Knowledge and Awareness of Tuberculosis among High School children in Vellore, India

**DOI:** 10.4103/0970-0218.62589

**Published:** 2010-01

**Authors:** Vijayaprasad Gopichandran, Priyankur Roy, Ashwin Sitaram, Karthick, KR John

**Affiliations:** Department of Community Medicine, Christian Medical College, Bagayam, Vellore - 632 002, India

## Introduction

Tuberculosis is a disease of great significance in India. With the advent of the HIV/AIDS epidemic the problem has been compounded. The Millenium Development Goal number 6, target 8, is ‘Halt and begin to reverse the incidence of TB by 2015’. In order to achieve this goal the World Health Organization (WHO) launched the Stop TB program; one of the important strategies of which was education and empowerment of communities. It is hypothesized that improving the knowledge and awareness about tuberculosis in school going children will spread the awareness in the general community. This study was done to assess the level of knowledge of high school children, about various aspects of tuberculosis, and to assess the impact of a simple educational intervention on the knowledge of the children, with regard to tuberculosis.

## Materials and Methods

The study subjects were students of the ninth grade of the Kaniyambadi Higher Secondary School, which has students from first grade to twelfth grade. The school is located in the project area of the Community Health and Development (CHAD) unit of the Christian Medical College, Vellore. There were a total of 470 students enrolled in the ninth grade class of the school of whom 375 participated in the study. The study was conducted during July 2008, when a questionnaire was distributed to the children for self-administration. It contained a total of 15 questions, to assess the knowledge of the students with regard to tuberculosis, on the following domains, namely, nature of the disease, disease transmission, signs and symptoms of tuberculosis, diagnosis, and its treatment and prevention. A 30-minute, audio-visual health education session was administered with the help of a PowerPoint presentation on tuberculosis, in the Tamil language, the local vernacular. Following this a printed handout was given, which contained these messages in the Frequently Asked Questions (FAQ) format, in Tamil. Subsequently an essay competition on tuberculosis awareness was conducted. The title for the essay competition was ‘Tuberculosis anywhere is tuberculosis everywhere’ — the theme of the world TB day, in the year 2008. After a week the same pre-test questionnaire was re-administered as a post test. Data were entered and analyzed in the EpiInfo 2002 software. Student paired T test was used to compare the pre-test and post-test responses.

## Results

The pre-test questionnaire was administered to 375 students. Out of the 375 students 104 did not answer the post-test questionnaire. Hence, 271 students were taken for the analysis. Of the 271 taken for analysis, 141 were boys and 130 were girls. The baseline knowledge of the students about tuberculosis is as tabulated in Table 1. About 77% of the students were aware that tuberculosis was caused by bacteria and 85% were aware that it could spread from person to person. Only 28% of the students knew that the treatment for tuberculosis was for six to nine months. Eighty percent of the students knew that cough and weight loss were the common symptoms of tuberculosis, but only 52% were aware that the sputum test was the diagnostic test of choice. The mean pre-test score was 7.05 (64%) and the mean post-test score was 9.15 (83.2%). The most significant change in knowledge was for the treatment of tuberculosis. This change in knowledge is depicted in Table 2.

**Table 1:** Baseline knowledge of the children about tuberculosis

Response	Percentage of students (%)
TB is caused by a bacteria	77.1
Lung is the most commonly affected organ	76.4
TB spreads from person to person	85.2
Tuberculosis is preventable	74.5
Tuberculosis treatment is free of cost	71.9
DOTS refers to a method of treatment of TB	27.3
Smoking causes TB	39.1
TB treatment is for 6–9 months	28.7
TB treatment is for 1 month	50.5
BCG vaccine prevents TB	75.2
Cough and weight loss are common symptoms of TB	80.4
Sputum AFB test is the diagnostic test of choice	52.1

TB= Tuberculosis, DOTS= Directly observed treatment short course, AFB= Acid fast bacilli

**Table 2:** Comparison of knowledge in various domains in pre-test and post-test – Student t test

Knowledge domain	Difference between mean	t score	Sigbificabce (*P* value)
About tuberculosis disease	−0.416	−10.663	0.00
Disease transmission	−0.505	−9.876	0.00
Symptoms	−0.110	−4.126	0.00
Diagnosis	−0.383	−11.600	0.00
Treatment	−0.826	−13.687	0.00

## Discussion

Important research has been done to assess the knowledge, attitudes, and practices with regard to tuberculosis, among different sections of the population in India. In a study among a north Indian urban community only 2.3% knew that TB was caused by a germ. Only 12.6% knew that the duration of treatment was six to eight months, and 1.7% knew about the preventive role of Bacillus Calmette-Guerin (BCG).(1) In the same north Indian community the impact of the Information Education Communication (IEC) campaign undertaken by the Government was studied. The core message regarding symptoms, diagnosis, treatment center, and free treatment was recalled correctly by 14.3, 44.5, 65.4, and 89.2%, respectively.(2) In another simple study in South India the impact of health education on the knowledge about tuberculosis, two years after the intervention, was studied. There was an overall increase in knowledge, between 18 and 58%, in various fields, at the end of two years.(3) This clearly showed that simple educational interventions could lead to significant changes in knowledge among the population. Young school going children are a very important target population for health education about tuberculosis. The importance of school going children is that they are at an impressionable age, and hence, powerful educational messages can be passed on to them. Moreover through school children parents can be reached and information can be disseminated among them. In one controlled study conducted in Western India, it was seen that the education of school children is a significant method of disseminating health information to parents who are illiterate and are not reached by the conventional methods of health education. School students between 11 and 15 years of age were given health education about leprosy. Subsequently the parents of these children were given a test on leprosy and their knowledge level was significantly high. About 18.4% of the parents reported that their children were the source of their information.(4)

In a study on health awareness among school children in Chandigarh, India, it was seen that 78.5% were aware about the modes of transmission of tuberculosis, 61% were aware about the signs and symptoms, but only 46.5% were aware about the various treatment aspects.(5) In the current study about 75 to 80% of the students knew about the communicability and modes of spread of tuberculosis. About 85% knew about the signs and symptoms. However, as in the previous studies the level of knowledge about diagnosis and treatment aspects was not good. The study also showed that a simple educational intervention such as a 30-minute, audio-visual presentation and distribution of pamphlets, significantly changed the way the students perceived the disease and its treatment. There was a statistically significant change in the level of knowledge in domains such as diagnosis and treatment of tuberculosis, which indicated that the empowerment of students could guide the community on treatment aspects. The study clearly demonstrated the impact of a simple, 30-minute, educational intervention, in the form of multimedia education, on the knowledge and awareness about tuberculosis among school children. The educational module could potentially be replicated in all schools in tuberculosis-endemic countries. School children could be empowered to be the flag bearers in the fight against tuberculosis.

## References

[1] Singh MM, Bano T, Pagare D, Sharma N, Devi R, Mehra M (2002). Knowledge and attitude towards tuberculosis in a slum community of Delhi. J Commun Dis.

[2] Sharma N, Taneja DK, Pagare D, Saha R, Vashist RP, Ingle GK (2005). The impact of an IEC campaign on tuberculosis awareness and health seeking behaviour in Delhi, India. Int J Tuberc Lung Dis.

[3] Thilakavathi S, Nirupama C, Rani B, Balambal R, Sundaram V, Ganapathy S (1999). Knowledge of tuberculosis in a south Indian rural community, initially and after health education. Indian J Tuberculosis.

[4] Bhore PD, Bhore CP, Powar S, Nade AL, Kartikeyan S, Chaturvedi RM (1992). Child-to-parent education: A pilot study. Indian J Lepr.

[5] Goel S, Singh A (2007). Health awareness of high school students. Indian J Community Med.

